# Supplementary material for: Identification of cellular signatures associated with chinese hamster ovary cell adaptation for secretion of antibodies
Source: Comput Struct Biotechnol J. 2024 Dec 10;27:17–31. doi: 10.1016/j.csbj.2024.12.006 (PMC11697065; doi:10.1016/j.csbj.2024.12.006)
Supplement: Supplementary file 1 — Supplementary material [file mmc1.docx]

**Supplementary Figure 1: Intracellular IgG Expression in Cell Pools.** Intracellular expression of heavy chain (HC) and light chain (LC) for No IgG control, mAb1, BisAb1, MonoAb1, BisAb2 and MonoAb2 pools as determined by flow cytometry. Quadrant 1 (Q1) corresponds to cells with only kappa LC expression, quadrant 2 (Q2) corresponds to double positive cells expressing both HC and kappa LC, quadrant 3 (Q3) corresponds to cells expressing only HC, quadrant 4 (Q4) corresponds to non-expressing cells. Q4 was determined based on naïve CHO cells stained with only secondary antibodies and unstained naïve CHO cells.

**Supplementary Figure 2: CHO Cell Pool Transcriptomic and Proteomic Analysis. A:** A bar chart indicating the number of significantly differentially expressed (DES) proteins and genes in cell pools as measured from TMT proteomics or RNA-seq. **B:** A heatmap showing significantly upregulated genes shared by all antibody producing cell pools when compared to the no IgG control with significantly expressed genes defined as adjusted-P value <0.05 and a fold change > 1.2. **C:** Volcano plot of differentially expressed genes from each cell pool compared to No IgG control. Adjusted-P value (FDR) <0.05 and fold change > 1.5 were used to define significantly expressed genes. Upregulated genes from each antibody producing cell pool are highlighted in red while downregulated genes from each antibody producing cell pool are highlighted in blue. Log2FC and -Log10 (P-adjusted value) were used to construct volcano plots.

**Supplementary Figure 3: Clonal CHO Cell Line Characterisation Data used for Selection of Clones for Omics Analysis.** Clonal cell line titre, growth (viable cell density, VCD), viability and cell specific productivity (qP) derived from day 7, 9 and 11 of the fed-batch process for mAb1 (**A**), BisAb1 (**B**), MonoAb1 (**C**), BisAb2 (**D**), MonoAb2 (**E**). Asterisk displayed on qP graphs denote clonal lines used for omics analysis with high qP clones in red and low qP clones in blue.

**Supplementary Figure 4: Intracellular IgG Expression in Clonal Cell Lines Used for Transcriptomic and Proteomic Analysis.** Intracellular expression of heavy chain (HC) and kappa light chain (LC) for mAb1, BisAb1, MonoAb1, BisAb2 and MonoAb2 expressing clonal cell lines as determined by flow cytometry. Quadrant 1 (Q1) corresponds to cells with only HC expression, quadrant 2 (Q2) corresponds to double positive cells expressing both HC and kappa LC, quadrant 3 (Q3) corresponds to cells expressing only Kappa LC, quadrant 4 (Q4) corresponds non-expressing cells.

**Supplementary Figure 5: Transcriptomic Analysis of Clonal CHO Cell Lines. A and B:** Heatmaps derived from RNA-seq data from clonal cell lines analysed on day 0 (D0) and day 6 (D6) of the fed-batch process. Data displayed by combining D0 and D6 (**A**) or displayed independently by day in culture (**B**). K-mean clustering was applied to data, enriched GO terms with biological process (BP) from each cluster are highlighted.

**Supplemental Figure 6: Correlation of Clonal CHO Cell Line Gene Copy Number, mRNA Abundance and Total Protein Abundance Separated According to Antibody Format. A and B:** Correlation of gene copy number and mRNA abundance for light chain (LC) (**A**) and heavy chain (HC) (**B**) for monoclonal antibodies. **C and D:** Correlation of gene copy number and mRNA abundance for LC (**C**) and HC (**D**) for bispecific antibodies. **E and F:** Correlation of mRNA abundance with protein abundance for LC (**E**) and HC (**F**) for monoclonal antibodies. **G and H:** Correlation of mRNA abundance with protein abundance for LC (**G**) and HC (**H**) for bispecific antibodies. Pearson correlation coefficient was used to test significance in correlation. P value *<0.05. **<0.01, ****<0.001

**Supplementary Figure 7: Gene Copy Number, mRNA Expression and Total Protein Abundances from Clonal Cell Lines. A:** Gene copy number of heavy chain (HC) and light chain (LC) for each clone by ddPCR at D0. **B:** mRNA expression of HC and LC for each clone derived from RNA-seq data at D0. **C:** Total protein abundance of HC and LC for each clone derived from TMT-proteomics at D0. **D**: Correlation of protein abundance of LC at D0 with the cell specific productivity (qP) from each clonal cell line.

**Supplementary Figure 8: Volcano Plots of Differentially Expressed Proteins Derived from TMT Proteomics. A-H:** Volcano plots of significant differentially expressed proteins from high secreting CHO clones compared to low secreting clones expressing mAb1, MonoAb2, MonoAb1 and BisAb1 on D0 (**A-D**) and D6 (**E-H**) with significantly upregulated proteins in red and significantly downregulated proteins in blue. The size of the dot represents the number of peptides detected. Significance is defined as Log2FC > 2 and adjusted-P value (FDR) <0.05.
