# Supplementary material for: Identification of cellular signatures associated with chinese hamster ovary cell adaptation for secretion of antibodies
Source: Comput Struct Biotechnol J. 2024 Dec 10;27:17–31. doi: 10.1016/j.csbj.2024.12.006 (PMC11697065; doi:10.1016/j.csbj.2024.12.006)

Supplementary Figure 1

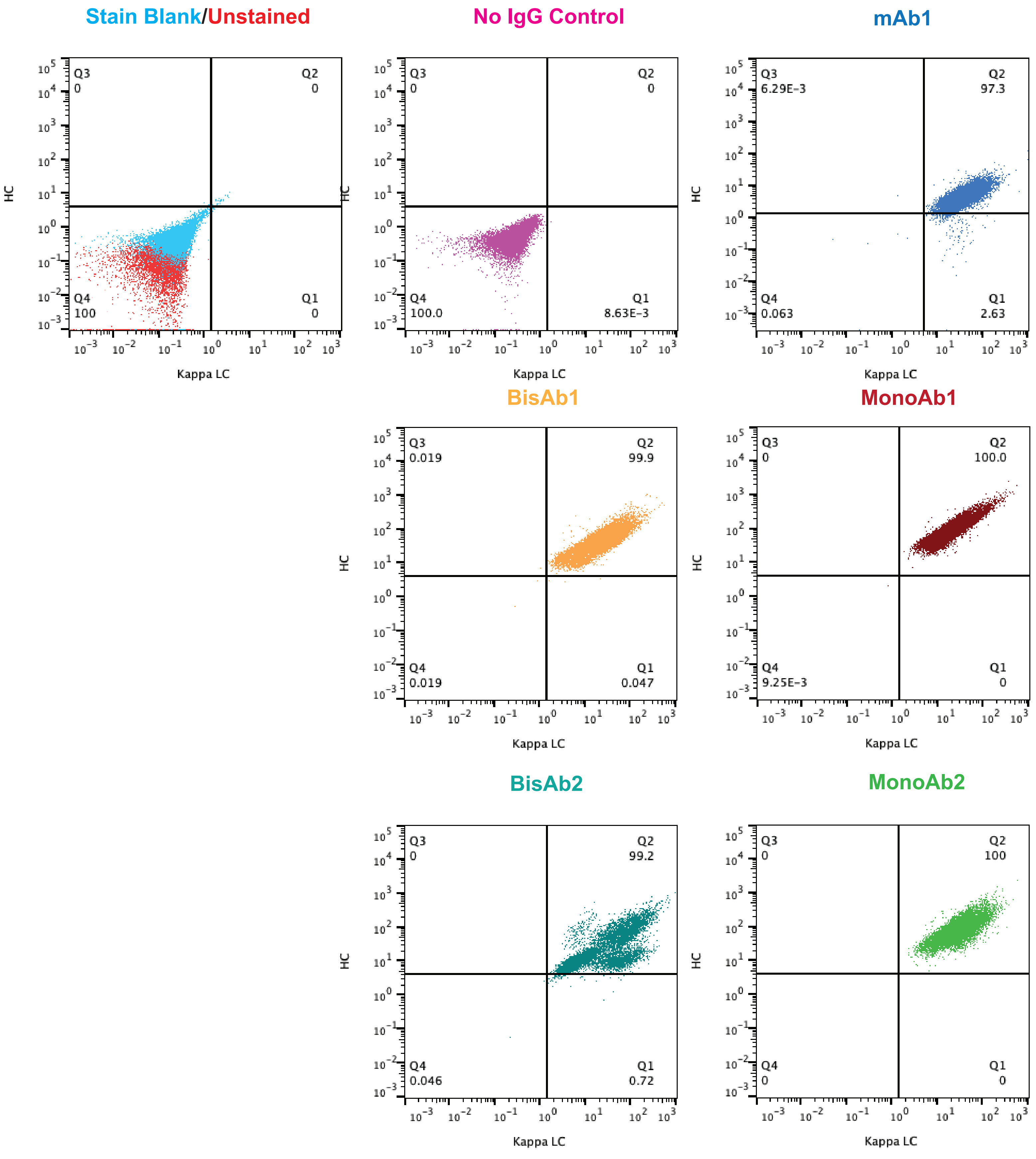

Supplementary Figure 2

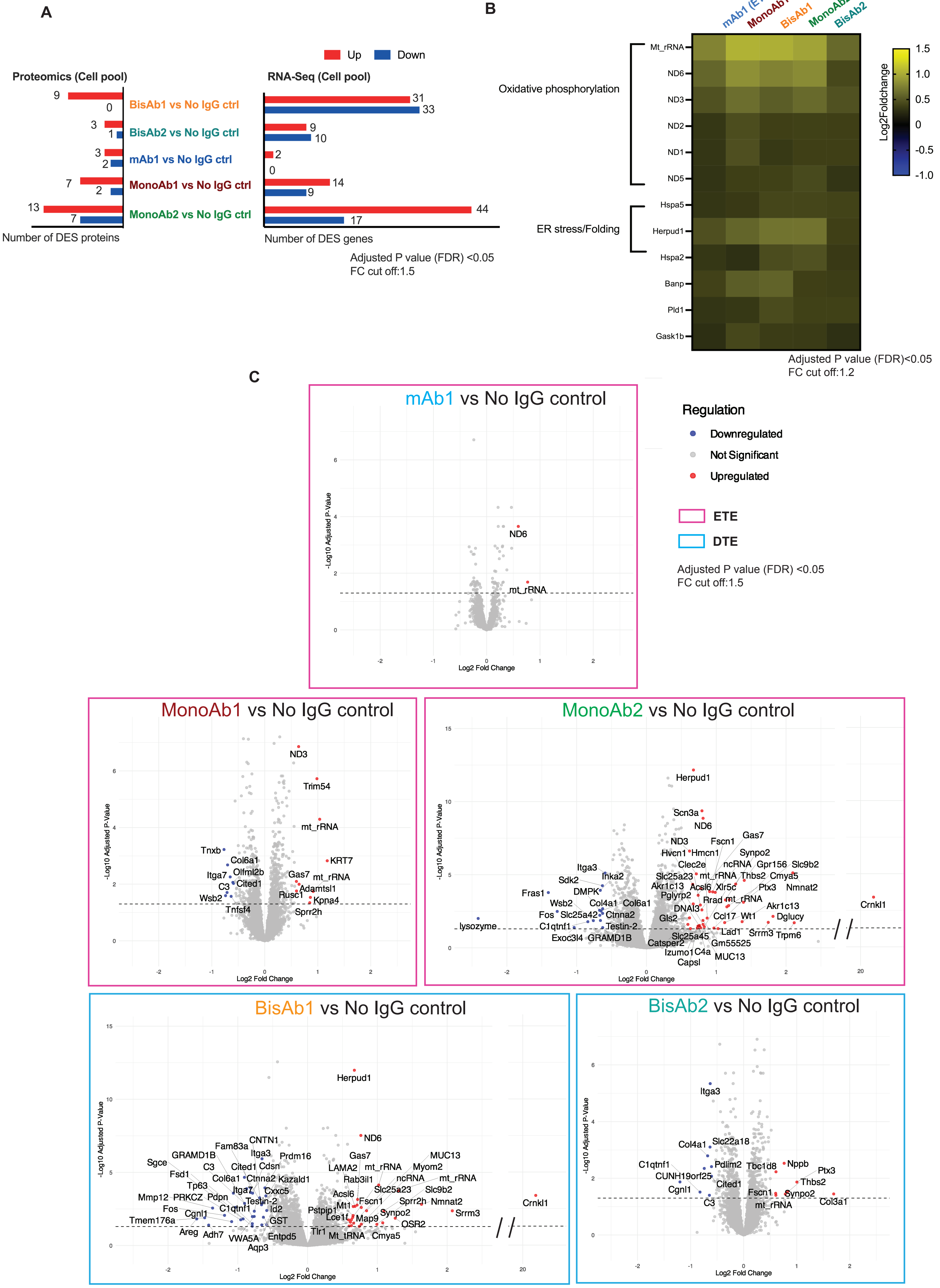

Supplementary Figure 3

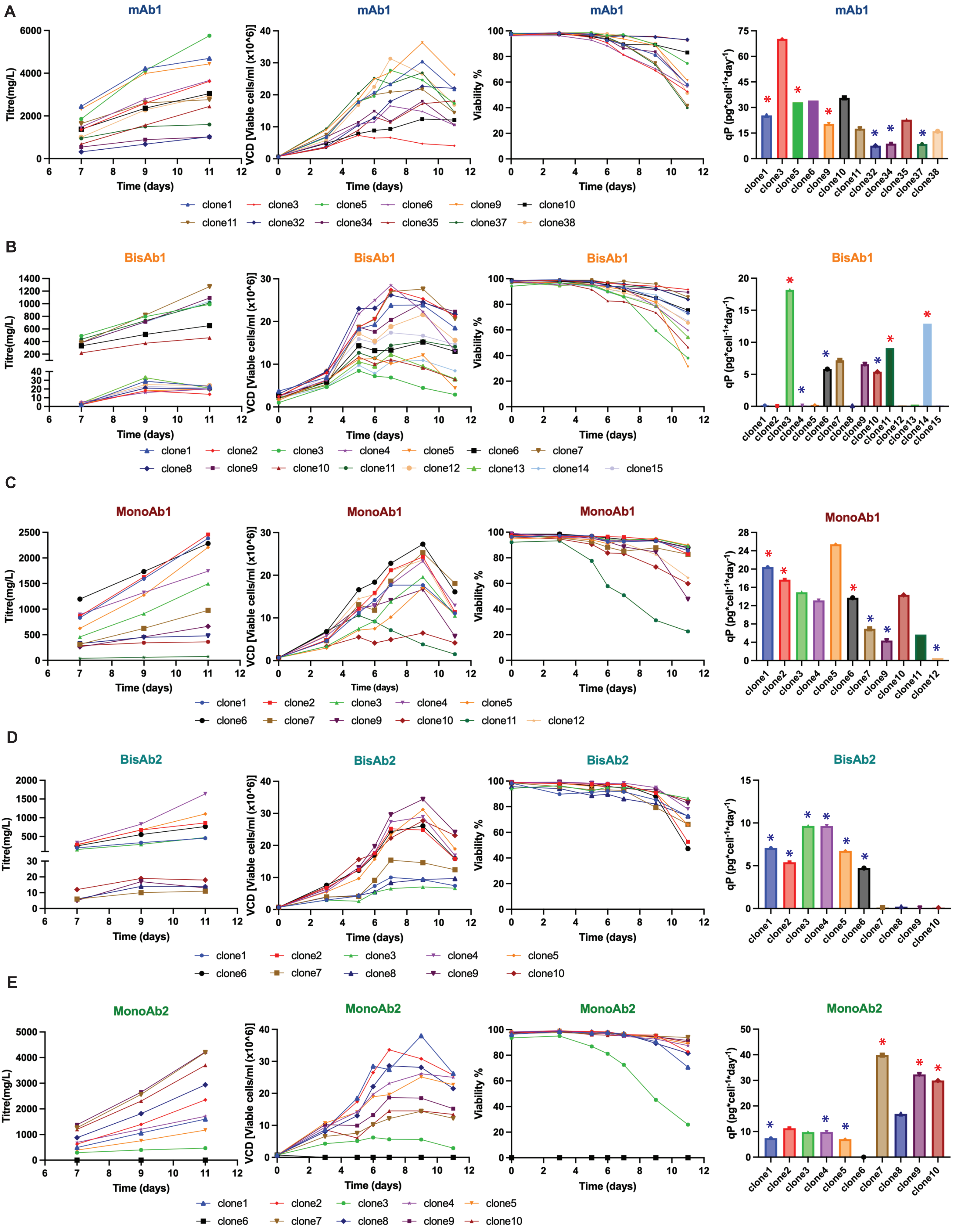

Supplementary Figure 4

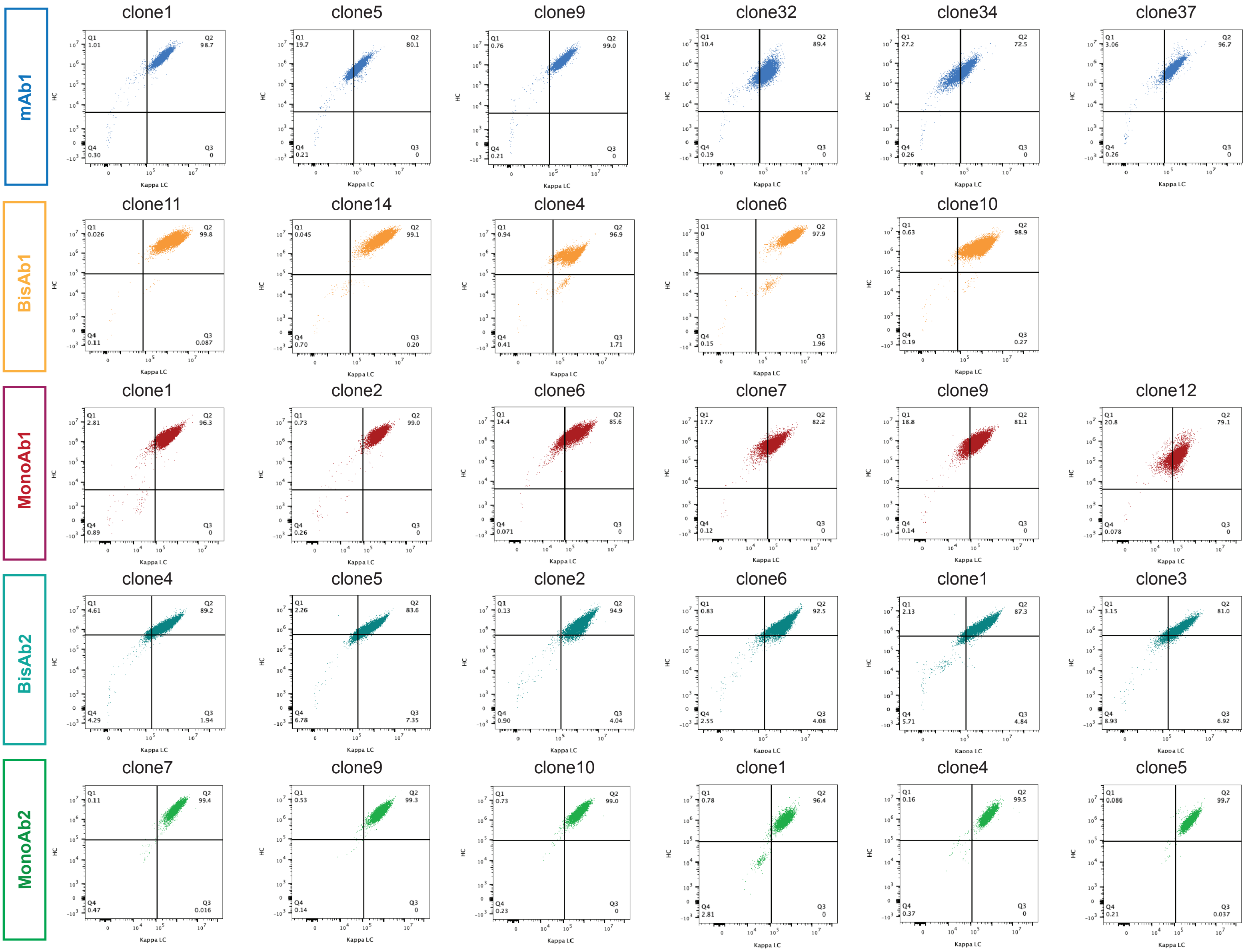

Supplementary Figure 5 RNA_seq

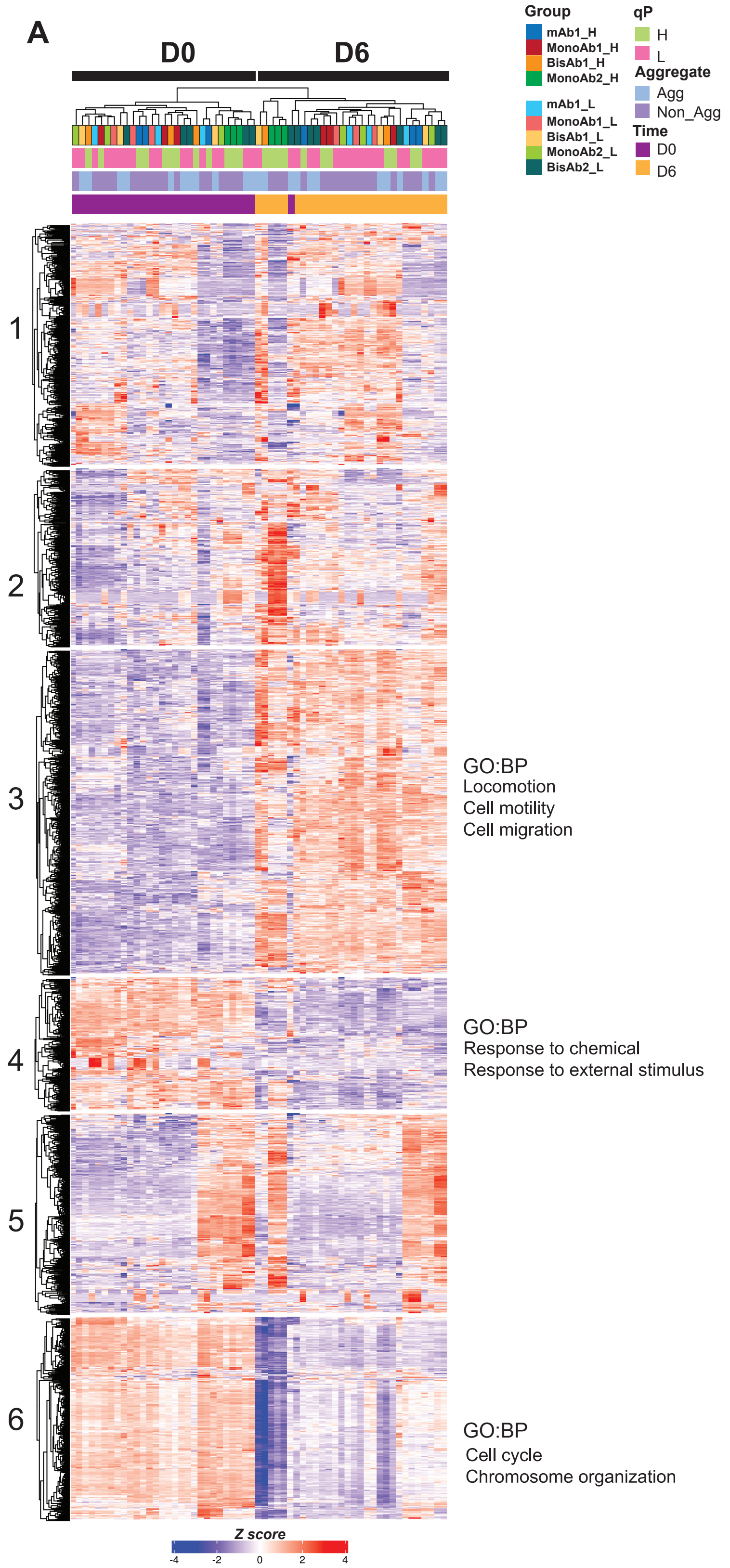

Supplementary Figure 6

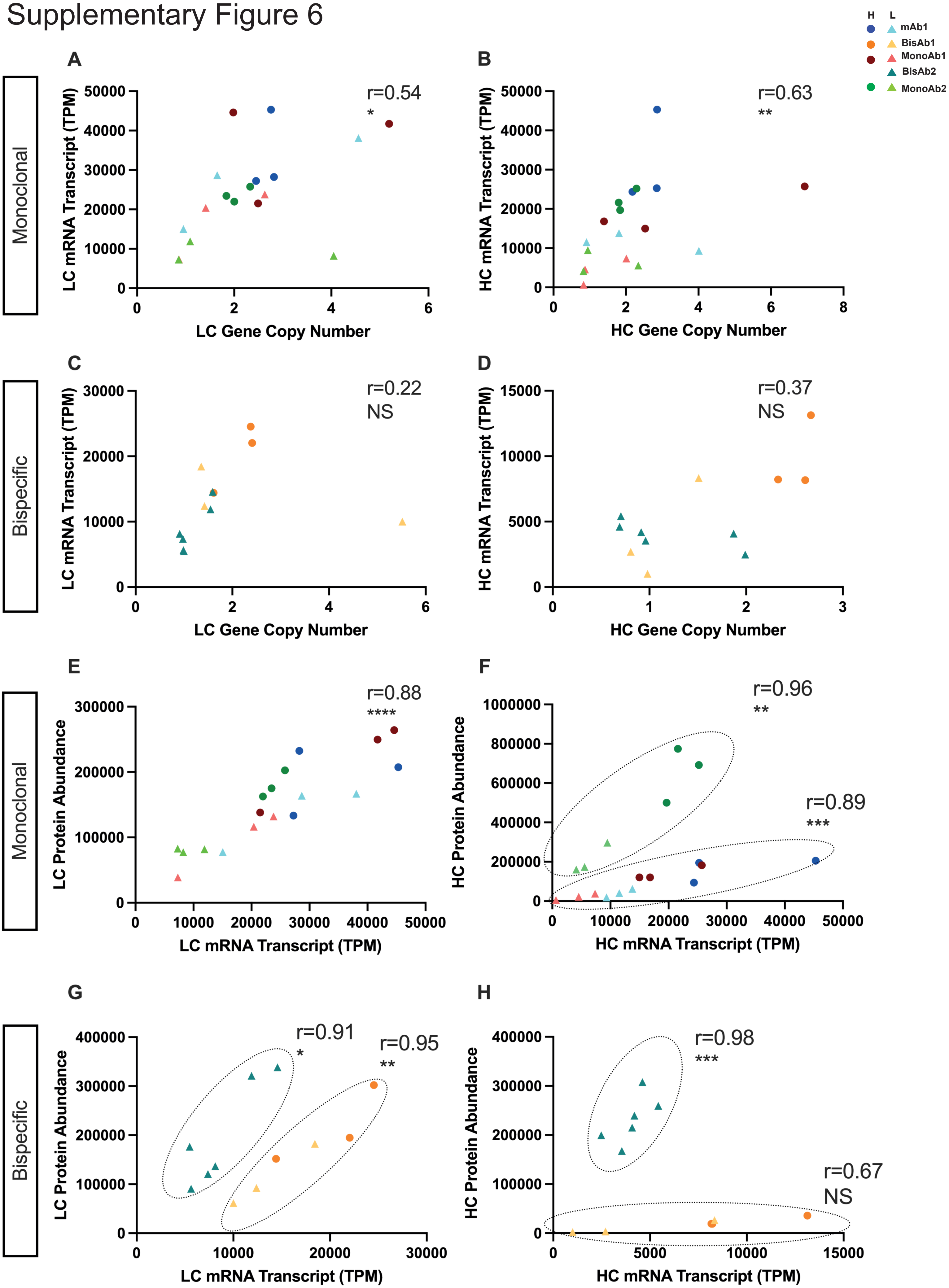

Supplementary Figure 7

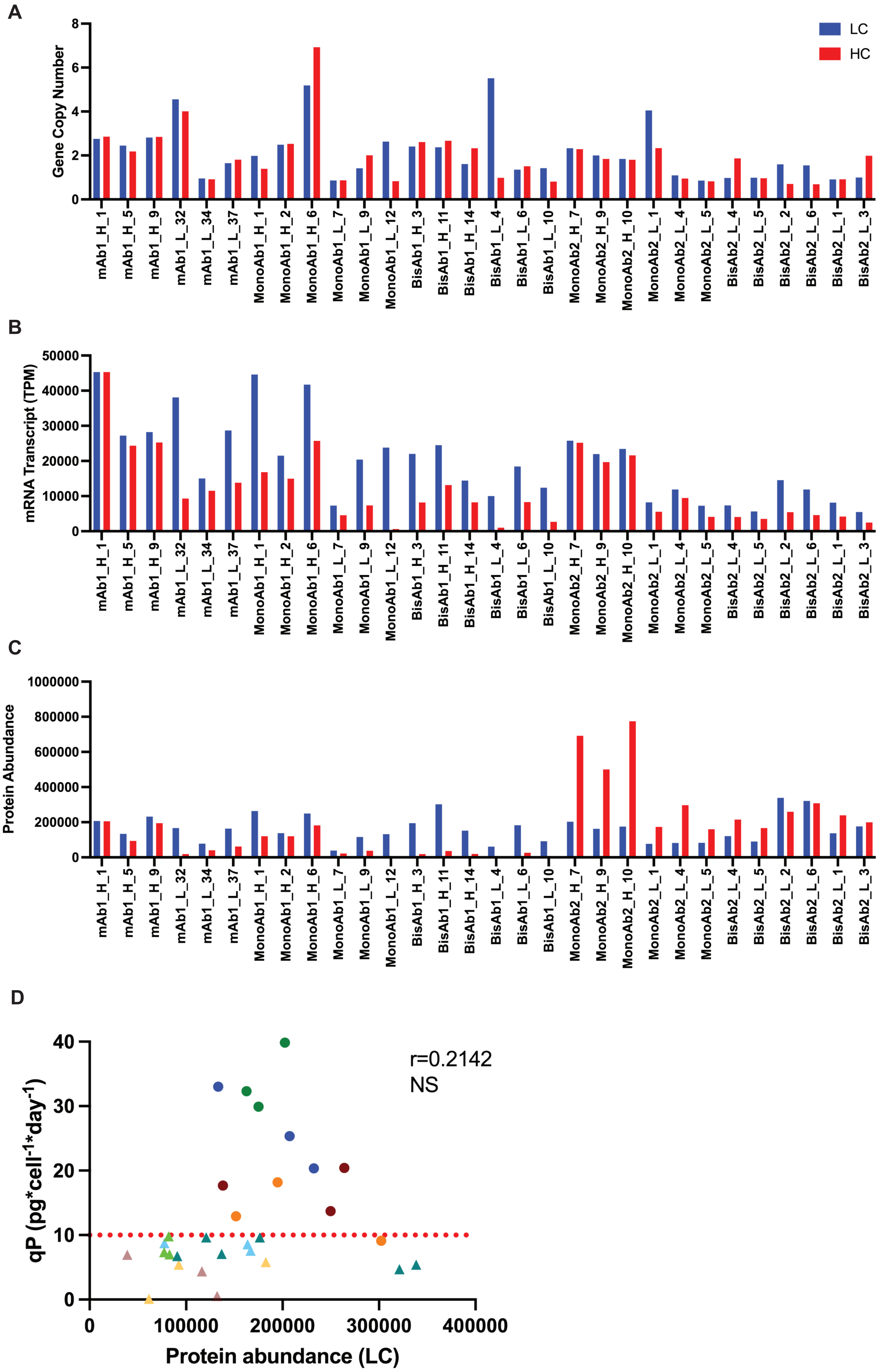

Supplementary Figure 8

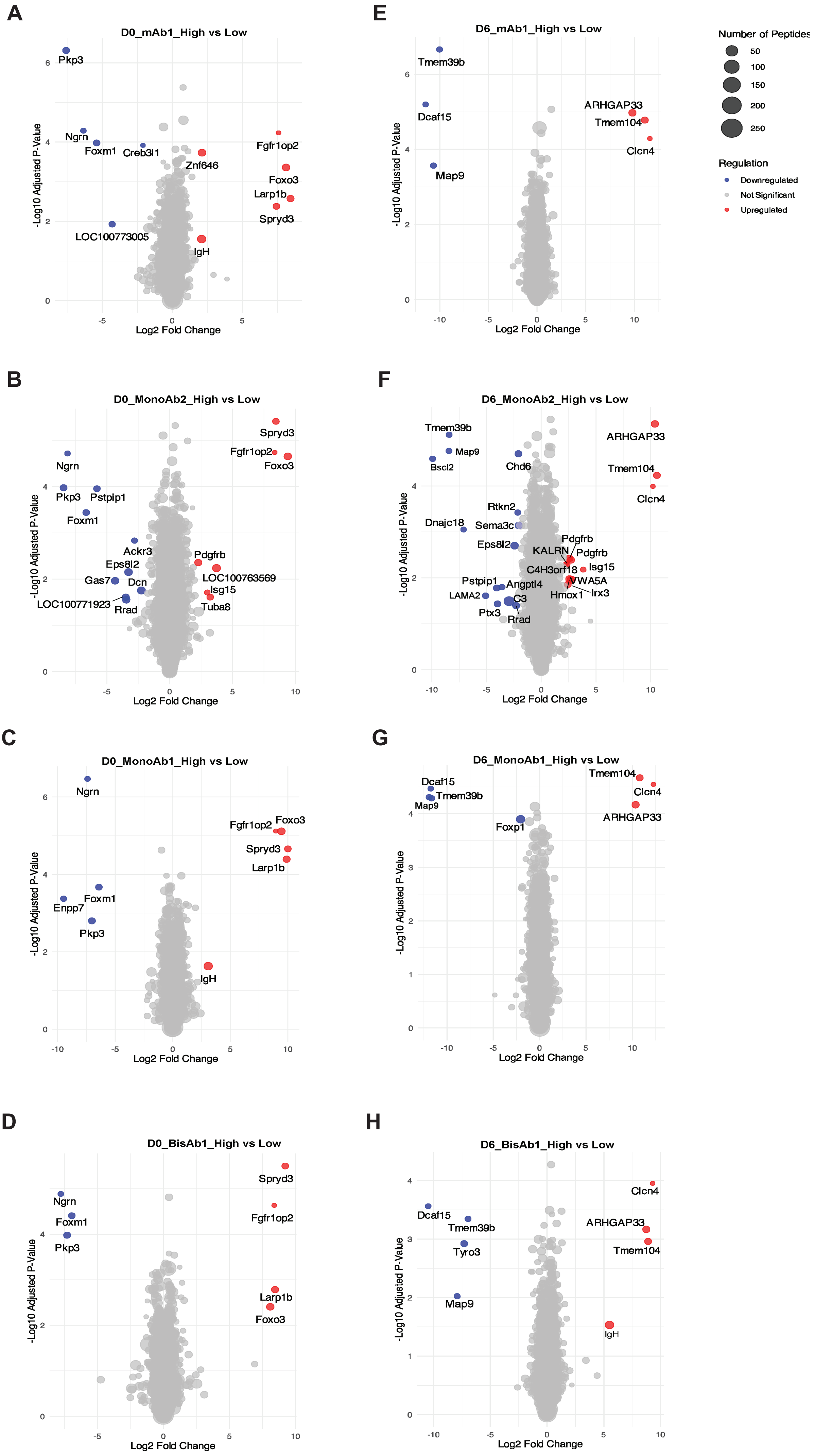

Supplement: Supplementary file 2 — Supplementary material [file mmc2.pdf]
